# Biosystematics of *Angulitermes dehraensis* in the Northwestern Indomalayan region by integrating morphometrics and distributional data with DNA barcoding

**DOI:** 10.3389/finsc.2025.1695789

**Published:** 2025-10-24

**Authors:** Xiuhua Lv, Xiaoxia Zhang, Rashid Azad, Maid Zaman

**Affiliations:** ^1^ Inner Mongolia Preschool Education College for the Nationalities, Ordos, China; ^2^ Jungar Banner Agriculture and Animal Husbandry Bureau, Ordos, China; ^3^ Department of Entomology, Faculty of Physical and Applied Sciences, The University of Haripur, Haripur, Khyber Pakhtunkhwa, Pakistan

**Keywords:** termites, *Angulitermes dehraensis*, morphometrics, distribution, DNA barcoding, COII

## Abstract

Termites are eusocial and economically important insects which are found in the world’s tropical regions as a harmful or beneficial organism. They play a dual role, both as pests damaging crops and urban structure and as an ecological engineer sustaining the ecosystem. Pakistan is part of the Indomalayan realm hosting diverse flora and fauna including termites; however, the status (diversity, distribution, feeding hosts, pest and non-pest) of the genus *Angulitermes* in the northwestern region (Khyber Pakhtunkhwa, Pakistan) has been largely neglected. Termite cultures were collected from diverse ecosystems, cleaned, and preserved in alcohol-filled vials for subsequent morphometric identification and DNA barcoding. Coordinates with relevant ecological data were also recorded. Soldiers were used for capturing refined images and morphometric identification through available literature, which resulted as an *Angulitermes dehraensis* and a new locality record. A revised and updated world’s species list for the genus was made along with the distribution map of this study via ArcGIS. The identified representative soldier’s leg was processed for mtDNA extraction followed by amplification and sequencing. The received sequence was subjected to BLASTn search, and only top 15 sequences via BLASTn search and then via manual search for taxon *Angulitermes* were retrieved from GenBank. Aligned and trimmed sequences were processed for phylogenetic tree (neighbor-joining and maximum-likelihood) construction and validation of understudy species sequence analogy. A novel sequence was submitted to GenBank for accession number (PX423737). Based on the available and recorded feeding host substrate data, it is a pest species which needs management.

## 1 Introduction

Termites are eusocial insects which are known for causing serious damages (1, 2) around the world. They are divided into seven families, and ~3% of the known species are responsible for the damages in buildings and crops (3). They live in colonies which consist of workers, soldiers, and reproductive caste. Workers are in abundance and are the only damage-causing caste. Soldiers supply defense to the colony and are fed by workers through trophallaxis (1). They are also used for the identification of species (4) including this study. The reproductive caste (King; Queen aided by primary or secondary reproductive) maintain the colony population.

Naturally, termites are present in diverse terrestrial ecosystems of the tropical regions and playing an important role of being either harmful (pests) to buildings, forests, and agriculture (5) or beneficial (ecosystem engineers) to the ecosystem (6). They attack humans’ belongings in urban structures, trees, and saplings in forests whereas they attack crops in the agroecosystem. There are also some species of low ecological interest in the subtropical environment which are causing losses in the urban environment (1), agriculture, and forestry. Losses caused by termites vary by region, species, and available food sources, with reported figures including worldwide losses of $40 billion, and regional losses such as $11 billion in the USA, $10–12 billion in Malaysia, $1.5 billion in Australia, $1 billion in China, $1 billion in Indonesia, $0.8–1 billion in Japan, $0.5 billion in France, $0.5 billion in Thailand, $100s of millions in the Philippines, $35.12 billion in India, $4 billion in Taiwan, $1 billion in China, and $0.5 billion in Fiji Islands (7). Despite of the damages caused, termites are also the ecological engineers and help in the decomposition of wood and animal dung, recycling of nutrients, improvement of soil’s stability, water-holding and absorbing capacity, vegetation and tree diversity, etc. They are also a source of food for the wildlife and help in pollination although rarely (8). The presence of termites in each habitat defines their ecological role; while they are considered pests in urban areas due to structural damage, they play a beneficial role in natural habitats by decomposing wood, dung, and other organic matter.

Termites are generally controlled by insecticide (dusts or solutions) application (2), but often management fails and damages are caused. Failure of control strategies may also include incorrect identification of the present harmful species leading to the application of hazardous insecticides. Correct identification is important for understanding the role of termites (economic and ecological) (9), but it always requires experts. Taxa having similar phenotypic traits (10, 11) and poor records (12) make identification more challenging. Addition of universally accepted molecular studies (13, 14) including DNA barcoding has revolutionized the insect’s systematics (15, 16) by providing a quick way of identification as compared with traditional taxonomy (17).

**Figure 1:**
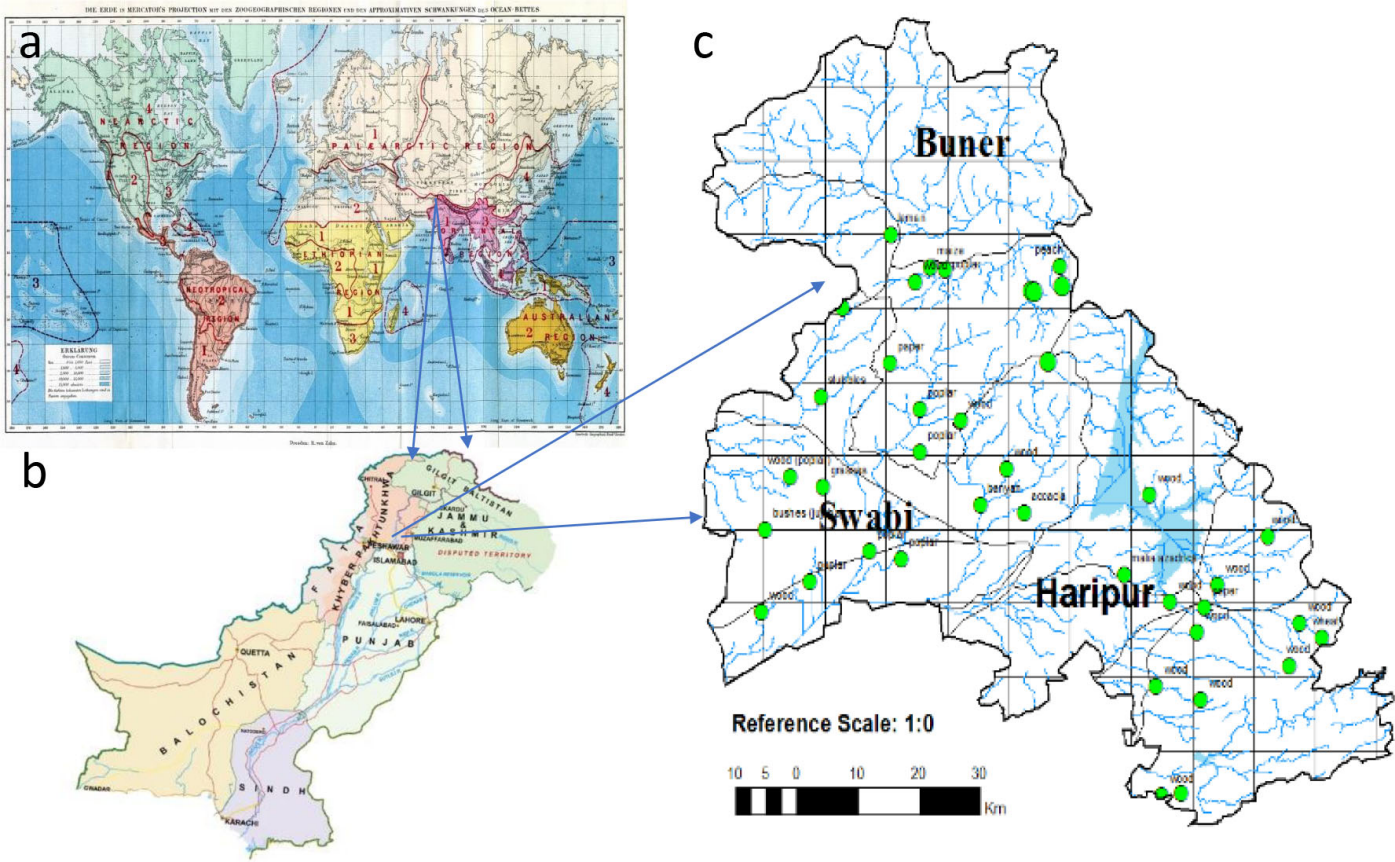
Genus *Angulitermes* sample’s collection from the selected study region of the Northwestern Indomalayan region (Pakistan; Khyber Pakhtunkhwa; Swabi, Buner and Haripur) **(a)** Wallace zoogeographical map; **(b)** map of Pakistan; **(c)** focused map of the study area.

The Indomalayan/Oriental region sprawls to the south of Himalayas in Southeast Asia, as shown in **Figure 1a** (18), having hot and humid climatic conditions. Pakistan (excluding Baluchistan) is part of the Indomalayan region with rich flora and fauna including termites, which is further divided into 10 agroecological zones by the Pakistan Agriculture Research Council (PARC) (19). According to Iqbal and Saeed (20), 11 out of 54 species (21) are economically important and belong to genera *Heterotermes*, *Coptotermes* (Rhinotermitidae), *Odontotermes*, and *Microtermes* (Termitidae) in Pakistan. Termites of Pakistan have been studied by Desneux (22, 23), Holmgren and Holmgren (24), Ahmad (25), Akhtar (26), Chaudry and Ahmad (27), and Nasir (28), excluding the northern Malakand (Buner), lower Hazara (Haripur), and eastern Mardan (Swabi) regions. This area belongs to three different agro-zones of Khyber Pakhtunkhwa (29), which was previously ignored for the termite’s faunal studies. No information was available on the species occurrence, distribution, and status (as a pest or non-pest) of termites from the genus *Angulitermes*, which could be utilized for designing any management or conservation strategy. Genus *Angulitermes* composed of 29 recorded species till date in which more than 70% species belong to the Indomalaya region (India, Myanmar, Pakistan, and Bangladesh) (**Table 1**). Therefore, the present study was conducted for studying the morphometrics, mapping, and distributional and feeding host substrate data with DNA barcoding of the termites from the genus *Angulitermes* in the northern Malakand (Buner), lower Hazara (Haripur), and eastern Mardan (Swabi) of Khyber Pakhtunkhwa-Pakistan, a part of the Northwestern Indomalaya region.

**Table 1:** Species composition of genus *Angulitermes* (30) (Termitidae: Promirotermitinae) from three major zoogeographic regions.

Species composition of genus *Angulitermes* (30)				
S. no	Species	Country	Zoogeographic region	Reference
1	*Angulitermes dehraensis*	Pakistan; India; Afghanistan	Indomalayan; Palearctic	
2	*Angulitermes hussaini*	Pakistan	Indomalayan	http://www.termitologia.net/catalog/gen.php?gen=Angulitermes&filtro=atual Retrieved on 20-08-2025
3	*Angulitermes punjabensis*	Pakistan	Indomalayan	
4	*Angulitermes acutus*	India	Indomalayan	
5	*Angulitermes akhorisainensis*	India	Indomalayan	
6	*Angulitermes bhagsunagensis*	India	Indomalayan	
7	*Angulitermes obtusus*	India	Indomalayan	
8	*Angulitermes fletcheri*	India	Indomalayan	
9	*Angulitermes jodhpurensis*	India	Indomalayan	
10	*Angulitermes kashmirensis*	India	Indomalayan	
11	*Angulitermes keralai*	India	Indomalayan	
12	*Angulitermes longifrons*	India	Indomalayan	
13	*Angulitermes mishrai*	India	Indomalayan	
14	*Angulitermes tilaki*	India	Indomalayan	
15	*Angulitermes ramanii*	India	Indomalayan	
16	*Angulitermes rathorai*	India	Indomalayan	
17	*Angulitermes resimus*	Myanmar	Indomalayan	
18	*Angulitermes paanensis*	Myanmar	Indomalayan	
19	*Angulitermes ceylonicus*	Sri Lanka	Indomalayan	
20	*Angulitermes emersoni*	Bangladesh	Indomalayan	
21	*Angulitermes nepalensis*	Nepal	Indomalayan	
22	*Angulitermes arabiae*	Saudi Arabia	Palearctic	
23	*Angulitermes asirensis*	Saudi Arabia	Palearctic	
24	*Angulitermes braunsi*	South Africa	Afrotropical	
25	*Angulitermes elsenburgi*	South Africa	Afrotropical	
26	*Angulitermes frontalis*	Botswana; South Africa	Afrotropical	
27	*Angulitermes nilensis*	Eritrea; Ethiopia; Kenya; Senegal; Sudan; Tanzania	Afrotropical	
28	*Angulitermes quadriceps*	Israel; Saudi Arabia; Yemen	Afrotropical; Palearctic	
29	*Angulitermes truncates*	Ghana; Kenya; Nigeria; Saudi Arabia; Senegal; Tanzania; Uganda; Zambia	Afrotropical; Palearctic	

## 2 Materials and methods

### 2.1 Study area and survey for culture collection

**Figure 2:**
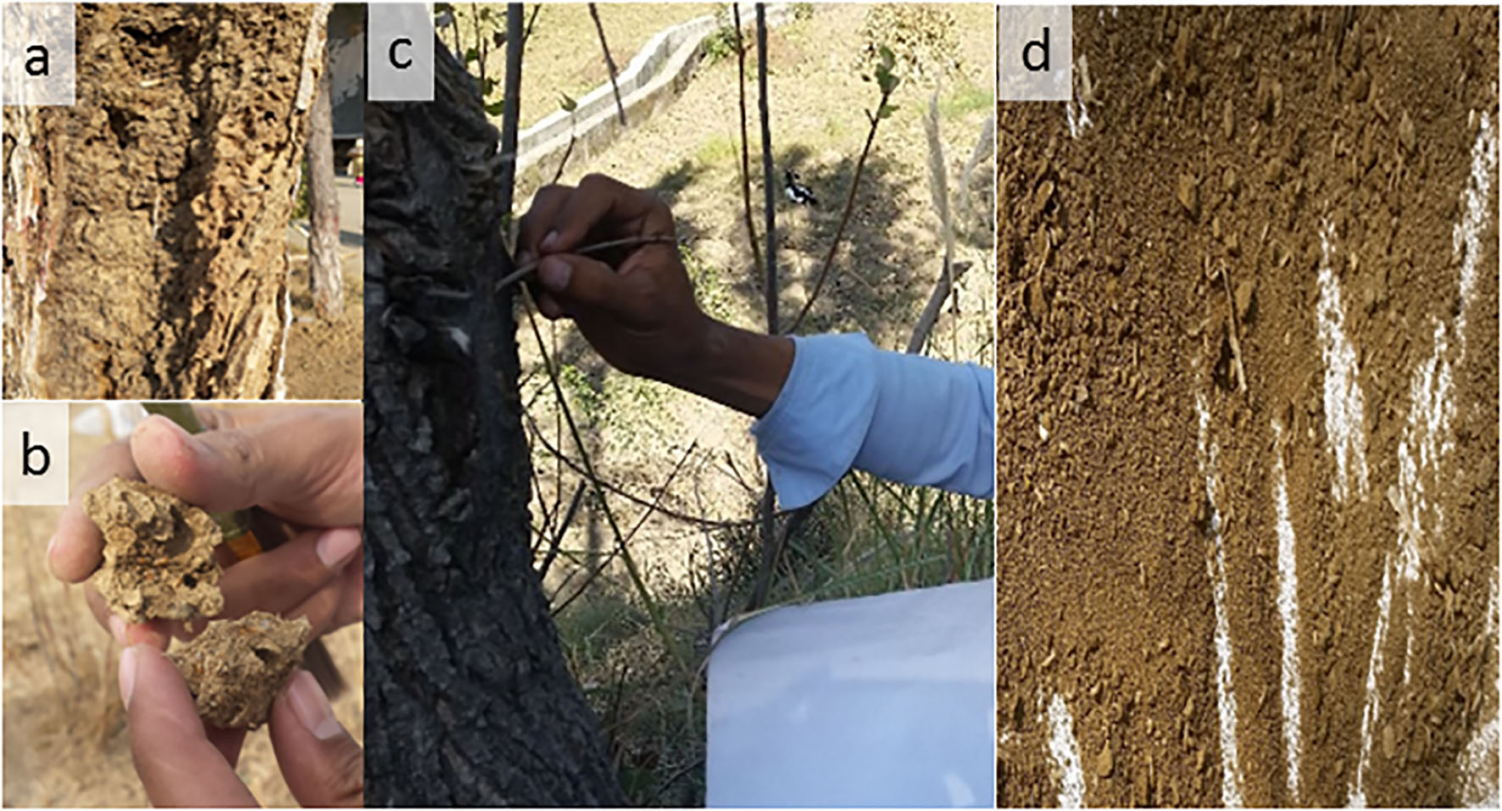
Termite’s collection protocol from the selected study region of Northwestern Indomalaya **(a)** termite galleries on a stem; **(b)** inspecting mud chunk for termite presence; **(c)** breaking mud galleries on the trunk for on spot collection; **(d)** termites with dirt after breaking mud galleries.

An intensive termite's culture collection survey was conducted for 3 years (in the summer season) in the Northwestern Indomalayan region (districts Swabi, Buner, and Haripur) (**Figures 1a–c**, **Supplementary Figure S1**) by following the modified method of Kaakeh (31) and Saha et al. (32). Visible mud galleries on the tree trunks (in forests/orchards) and walls (in buildings) were broken just above the ground, and termites running in the broken galleries were collected in a paper funnel with a gentle swipe of a camel brush (**Figures 2a–c**). The funnel was attached to either a collecting vial (containing alcohol 75% or 99%) or a wet blotting paper in case of any attached dirt. The termites became stuck to the wet blotting paper, and dirt was removed (**Figure 2d**) by a gentle tap. Cleaned and sorted specimens were stored in the tagged vials for morphometric identification and DNA barcoding. Sampling point coordinates (via GPS device eTrex 10), feeding host substrate, and other relevant ecological data (habitat type, elevation, canopy covered) were also recorded.

### 2.2 Termite’s morphometric identification

Randomly selected soldiers were used for the identification of 13 morphological characters and five indices (**Table 2**) by following Chaudry and Ahmad (27) and Chhotani (33). All measurements were taken (in millimeters) under a trinocular stereo-zoom microscope (Nikon 745T) with a mounted Nikon FSi2 digital camera for capturing pictures. The captured pictures were stacked by Helicon Focus 6.0 (Helicon Soft Ltd, Ukraine, 2000-2022) and further refined by Adobe Photoshop (Version CS6 13.1.2 San Jose, CA, USA, 2017). Means for all characters and indices were calculated in Microsoft Excel 365 (34).

**Table 2:** List of morphological characters and indices for the morphometric identification of *Angulitermes* species.

S. no.	Morphological characters/indices for genus *Angulitermes* identification
1	Length of frontal projection
2	Length of left mandible from the base
3	Length of head to side base of mandible
4	Head length with mandible
5	Head width max
6	Width of pronotum
7	Length of pronotum
8	Postmentum max width
9	Postmentum length
10	Postmentum medium waist
11	Width of labrum
12	Length of labrum
13	Total body length
14	Mandible head index (length of mandible/length of head)
15	Head index (width/length)
16	Mandible head index (length of mandible/length of head)
17	Pronotum index (pronotum length/pronotum width)
18	Pronotum head index (minimum width of pronotum/maximum width of head)

### 2.3 Distributional data

The sampling site’s (via GPS device eTrex 10) coordinates were recorded during surveys, which were then used for the construction of maps in ArcGIS 10.0 (35).

### 2.4 Molecular analysis

An identified representative soldier was washed with distilled water and allowed to air-dry. Then, the broken hind leg was ground with the pipette tip for DNA extraction followed by amplification (36) (**Supplementary Table S1**). For sequencing, 15µl of the extracted DNA was added to washed and sterile tubes having 2µl reverse and 2µl forward primers. The prepared samples were sent for Sanger sequencing (to Eurofins, Denmark; https://www.eurofins.com/).

For validating sequence analogy, received sequences were processed in BLASTn (NCBI) search for retrieving correct matches (37). Based on percent query covered, percent identity, bit cover, and relevant taxon match, only top-matched BLASTn (NCBI) sequences were retrieved for phylogenetic tree construction (**Supplementary Table S4**). Reference sequences were also searched manually for taxon “*Angulitermes*” (38). Then, all retrieved sequences were aligned in MEGA 6.0 by using ClustalW (39) and both ends were trimmed for removing any ambiguities. Neighbor-joining and maximum likelihood methods were employed for phylogenetic tree construction in MEGA 6.0 (40). Resulted novel sequences were submitted to GenBank for accession number.

## 3 Results and discussion

Termites from genus *Angulitermes* (30) are distinct and easily identified by the soldier’s frontal projection as compared with other termites. It is recorded from the Indomalayan, Afrotropical, and Palearctic zoogeographic regions with 29 known species in which 21 species are reported for the Indomalayan region (India, Nepal, Myanmar, Pakistan, and Bangladesh), making it a diverse region for this genus (**Table 1**). There are only three species, namely, *Angulitermes dehraensis* (Gardner) (41), *A. hussaini* (Ahmad), and *A. punjabensis* (Akhtar) reported from Pakistan (27) with unknown status (pest or non pest). In this study, *A. dehraensis* is recorded as a new locality record from a sub-hilly terrain (**Supplementary Table S3**).

### 3.1 Angulitermes dehraensis

#### 3.1.1 Diagnostic characters of soldier caste

**Figure 3:**
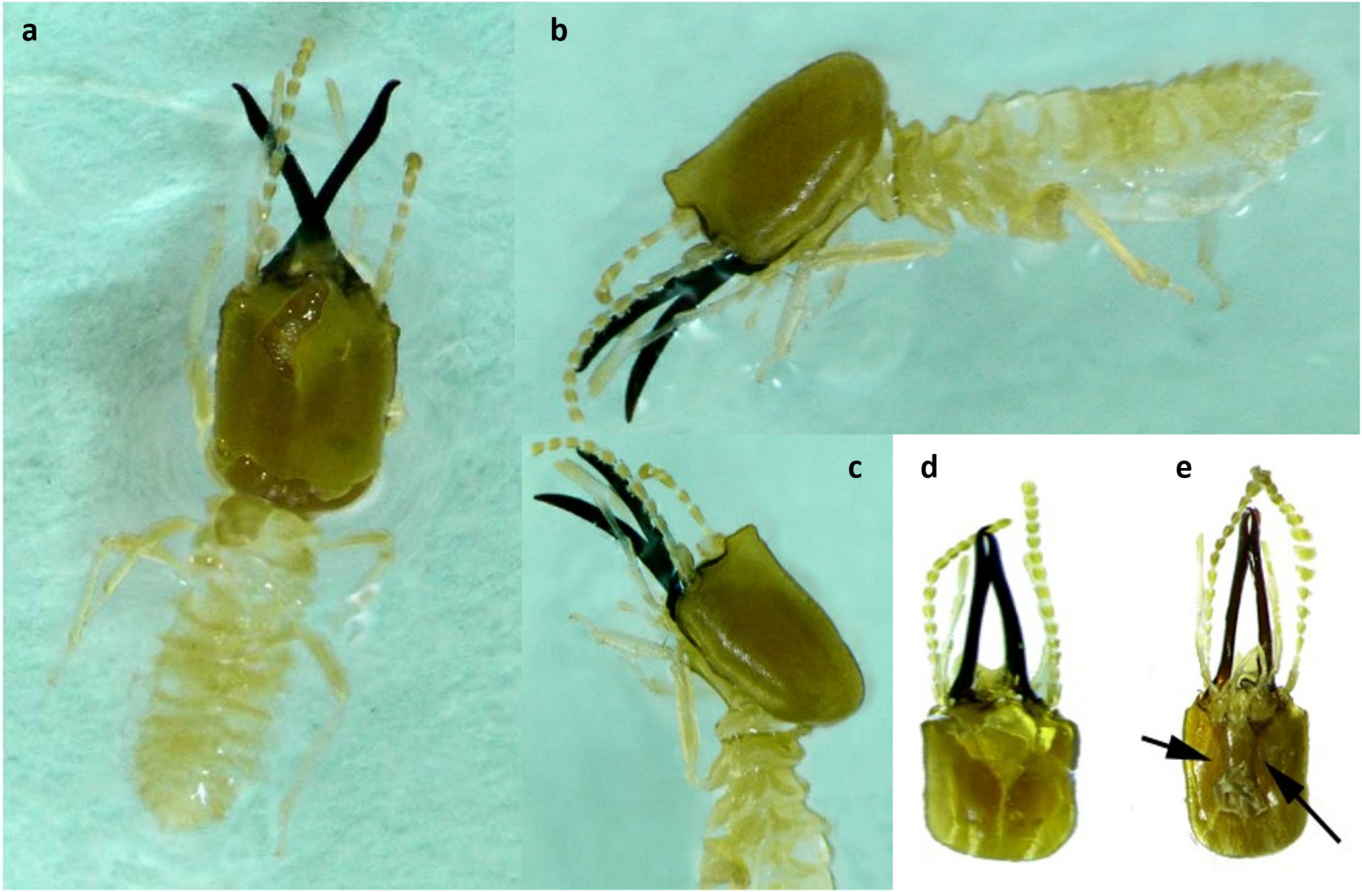
**(a–e)** Different diagnostic characters of *Angulitermes dehraensis* soldiers **(a)** dorsal view of the soldier; **(b)** side view of the soldier; **(c)** head region (fontanelle) of the soldier; **(d)** dorsal view of the head; **(e)** ventral view of the head (arrow = postmentum).

Head’s capsule, antenna, legs, body, and mandibles are of yellowish, fawn, straw yellow, and reddish-brown in color, respectively. Relatively the head is less hairy as compared with the body and frontal projection which are hairier. Antennae are of 14 segments in which segment 3 is longer than segment 2 whereas segment 4 is the shortest (**Figures 3a–e**). Other measurements include the following:

Mandibles = 1.34-1.45

Total body length= 3.90-4.60

Labrum; length= 0.33, width= 0.30

Pronotum; length = 0.23, width = 0.50

Length of frontal projection= 0.10-0.13

Postmentum; length= 0.43-0.40, max. width = 0.30, width at waist = 0.25

Index: Left mandible-length/head-length to base of mandibles= 1.16-1.25

All measurements are in millimeters. Details on characters are also available at Chhotani (33).

##### 3.1.1.1 Morphometrics

**Figure 4:**
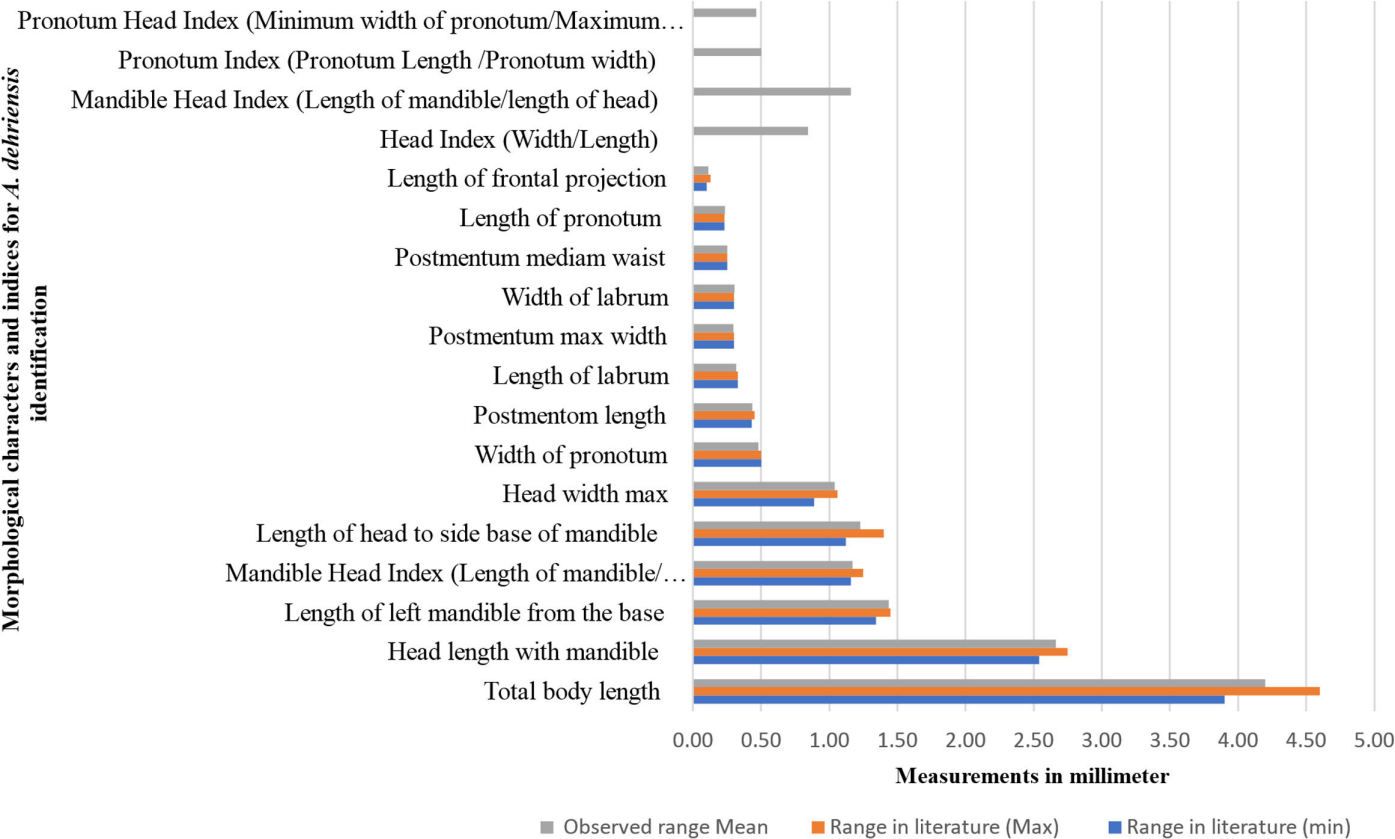
Morphometrics comparison of *Angulitermes dehraensis* with the measurement ranges available in the literature for species confirmation.

Measurements for the 13 characters and five indices were recorded and then compared with the ranges in literature (33) for the confirmation of understudy species morphometrics (**Figure 4**). Top four indices are adopted from other species and reported for the first time for *A. dehraensis* with no available measurement ranges in the literature (**Supplementary Table S2**). All the observed measurements are falling within the available morphometric ranges, which confirms that the specimens are of *A. dehraensis*, a species of genus *Angulitermes* (**Figure 4**).

### 3.2 Distribution and habitat preference

Chhotani (33) Rathore and Bhattacharyya (42); and Krishna et al. (43) reported *A. dehraensis* from India (Indomalayan region) and Afghanistan (Palearctic region) whereas Chaudry and Ahmad (27) and Ahmad and Akhtar (44) reported it from Pirwala forest, Chattarbagh, Chinari, Hangu, Parachinar, and Bara in Pakistan (Indomalayan region).

**Figure 5:**
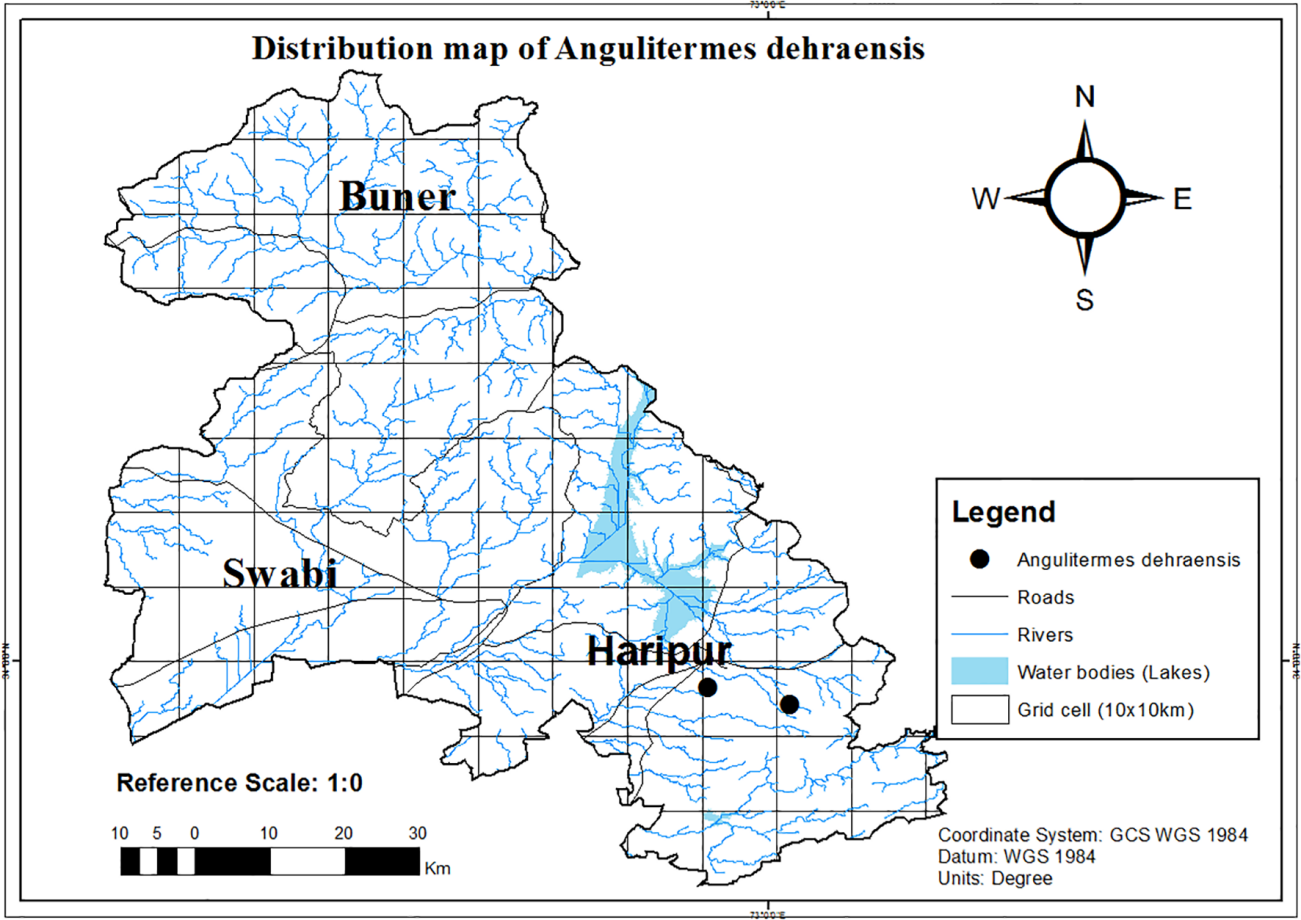
Distribution map of *Angulitermes dehraensis* in the selected study region of Khyber Pakhtunkhwa-Pakistan, a part of the Northwestern Indomalayan region.

During this study, culture collection was done from agroecosystem, forest, and urban ecosystem. However, *A. dehraensis* was only reported from guava orchard (agricultural ecosystem) as a new locality record for Haripur (**Figure 5**, **Supplementary Table S3**) and was found absent in structures/buildings and forests. This is a rare species which is found in the hilly areas of Pakistan.

### 3.3 Feeding host substrate

*A. dehraensis* was found feeding on the twigs, stumps, stem, date tree, and cow dung (27, 42, 44). However, in this study, it was feeding on the humus inside a hollow guava tree, making the tree survival at risk. Keeping this in view, this is a pest species of trees (dead/alive) which needs management.

### 3.4 DNA barcoding

For phylogenetic analysis, only top 15 sequences based on percent query covered, percent identity, bit cover, and relevant taxon match from GenBank were selected (**Table 3**). Then, GenBank was manually searched for the taxon/species name “*Angulitermes dehraensis*” and reference sequence, resulting DQ442073.1 as a relevant sequence with no reference sequence. Taxonomically, all the matched-up sequences were from four genera of family Termitidae. In the absence of a reference sequence, DQ442073.1 was used as a standard for analogy validation (**Supplementary Figure S2**) showing 99.02% homology with the understudy sequence.

**Table 3:** List of the top 15 GenBank retrieved sequences for *Angulitermes* species analogy validation.

S. no.	Accession number	Species name	Genus	Reference
1	MZ008538.1	*Amitermes* sp.	*Amitermes*	https://blast.ncbi.nlm.nih.gov/Blast.cgi# Retrieved on 15-08-2025
2	MZ008530.1	*Amitermes* sp.		
3	KY224528.1	*Amitermes* sp.		
4	OQ078686.1	*Amitermes unidentatus*		
5	OQ078687.1	*Amitermes unidentatus*		
6	PV057196.1	*Angulitermes* sp.	*Angulitermes*	
7	DQ442073.1	*Angulitermes* sp.		
8	DQ442165.1	*Microcerotermes dubius*	*Microcerotermes*	
9	OQ130293.1	*Microcerotermes subtilis*		
10	OQ130294.1	*Microcerotermes subtilis*		
11	DQ442228.1	*Promirotermes cf. redundans*	*Promirotermes*	
12	KP026266.1	*Promirotermes redundans*		
13	OL875039.1	*Promirotermes redundans*		
14	DQ442229.1	*Promirotermes redundans*		
15	KY224554.1	*Promirotermes* sp.		

#### 3.4.1 Neighbor-joining (N-J) tree

**Figure 6:**
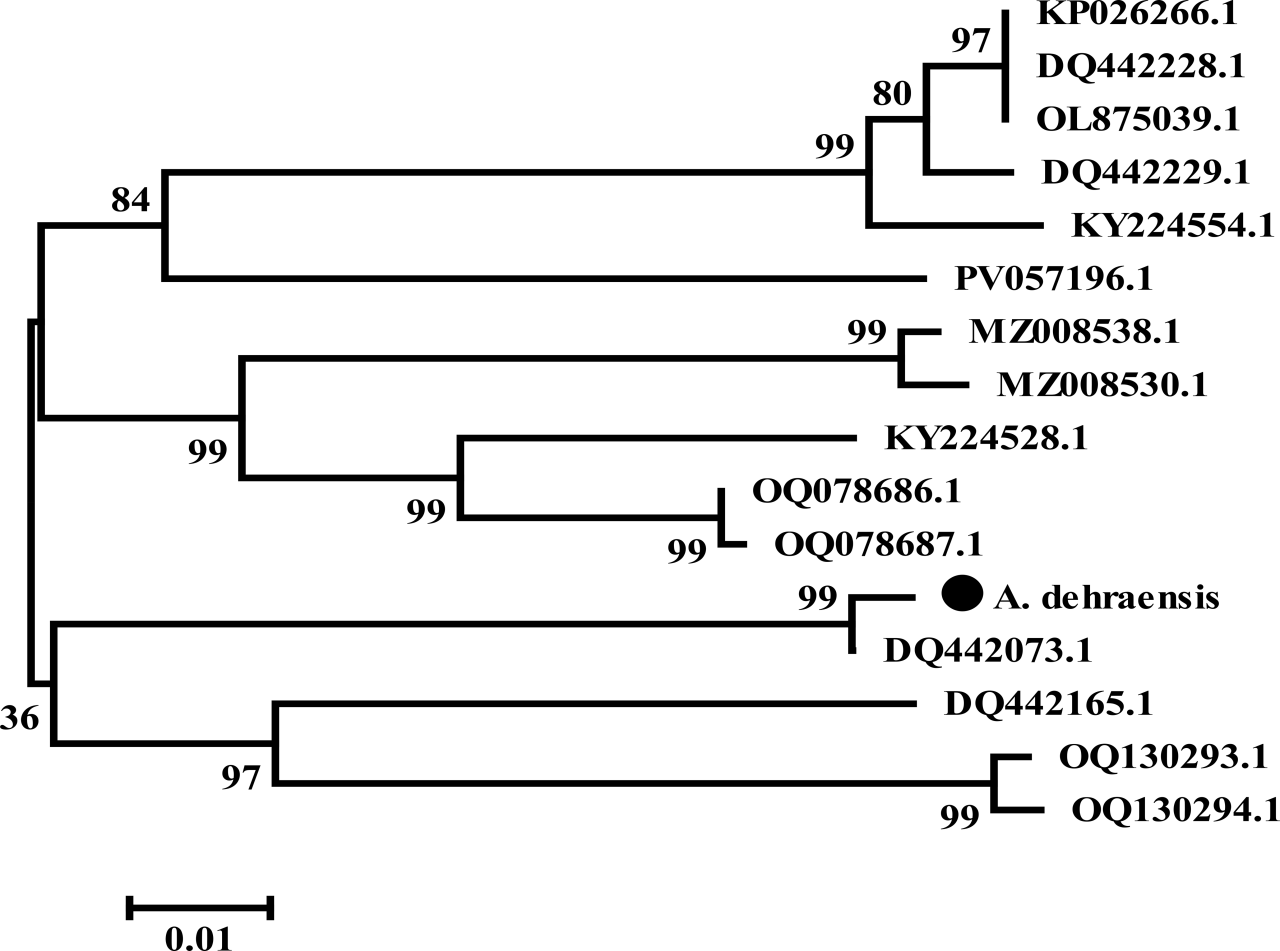
Unrooted neighbor-joining tree of *Angulitermes dehraensis* collected from the selected studied area of Khyber-Pakhtunkhwa, Pakistan, a part of the northwestern Indomalaya region. (● = under study sequence).

An N-J tree was constructed in MEGA (45) via the neighbor-joining method (46) and resulted into an unrooted tree consisting of two main branches. Branch 1 consists of 11 sequences, which is further branched for the sister species of the genera *Angulitermes, Promirotermes*, and *Amitermes*, whereas branch 2 consists of five sequences for the remaining sister species of the genera *Angulitermes* and *Microcerotermes*, which is further branched. Subbranch 1 shows the understudy sequence denoted by “●” with DQ442073.1, whereas subbranch 2 shows the remaining three sequences as shown in **Figure 6** (details on analysis are available at supplementary data N-J tree).

#### 3.4.2 Maximum likelihood (M-L) tree

**Figure 7:**
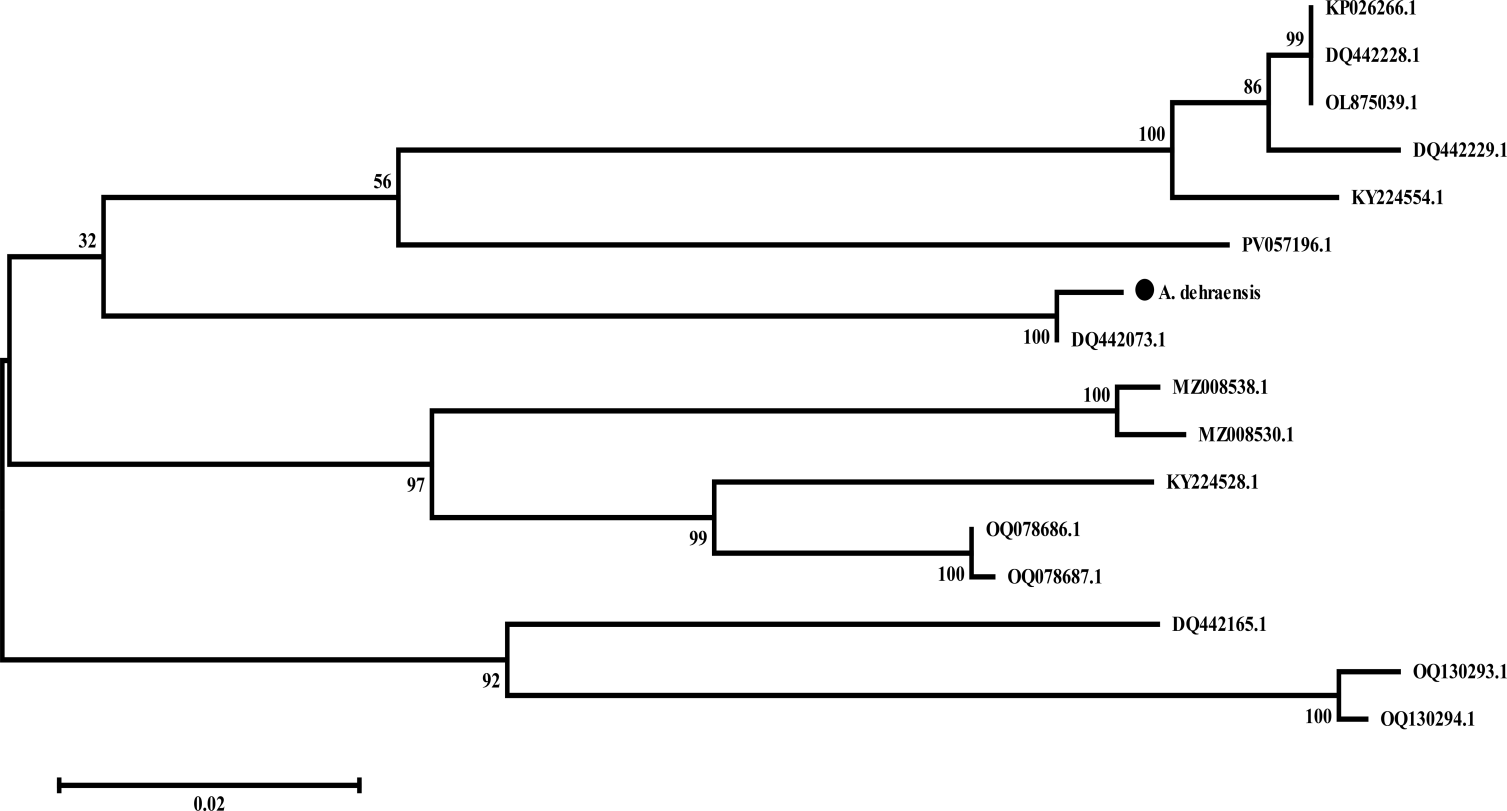
Unrooted maximum-likelihood tree of *Angulitermes dehraensis* collected from the selected studied area of Khyber-Pakhtunkhwa-Pakistan, a part of the Northwestern Indomalayan region. (● = under study sequence).

An M-L tree was constructed in MEGA (45) via the Tamura-Nei model (47) and resulted into an unrooted tree consisting of two main branches. Main branch 1 consists of 13 sequences of genera *Angulitermes, Promirotermes*, and *Amitermes* with two subbranches. Subbranch 1 is further divided into two more smaller branches; one contains eight accessions of the sister species along with the understudy sequence denoted by “●” with DQ442073.1, whereas the other contains the remaining five accessions. Main branch 2 represents the three sequences of genera *Microcerotermes* as shown in the **Figure 7** (details on analysis are available at supplementary data M-L tree).

Hebert et al. (48), Austen et al. (49), Firouzabadi et al. (50), and Adetitun (51) stated that there could be genetic diversity of 0.0%–0.51% among intraspecies whereas 97% among interspecies. In this study, the understudy sequence matched 99.02% (**Supplementary Figure S2**) with sequence DQ442073.1 (*Angulitermes* sp.), which is an incomplete taxon. Divergence of 0.98% with the understudy sequence is greater than the range 0.0%–0.51%, which indicates the novelty of understudy sequence, also supported by N-J and M-L tree analyses as shown in **Figures 6**, **7**. The reference sequence curated by NCBI is absent for the genus *Angulitermes*, making it a poorly recorded taxon. Clustering of sequence PV057196.1 (*Angulitermes* sp.) with other sister species and homology of 88% (**Supplementary Figure S3**) with the understudy sequence reveals wrong taxon naming.

## 4 Remark on specie status

*A. dehraensis* is a valid species and reported by several researchers including Chaudry and Ahmad (27), and Ahmad and Akhtar (44) for Indomalaya and Palearctic regions. It is a rare species which was collected from two localities of a sub-hilly terrain in a 3-year study. In this study, it was found feeding on the humus in a hollow guava tree trunk sharing the living space with the *Odontotermes obesus* species (33, 36). Based on the available and collected data of various feeding host substrate in horticultural ecosystems of the Indomalayan region, it is declared as a pest species of termites (43, Vol. 1, p. 145), which needs management.

Genus *Angulitermes* is poorly recorded in GenBank with no COII sequence data for *A. dehraensis* or any other species of the genus. Thus, a novel COII sequence of *A. dehraensis* is submitted to GenBank for accession number. It will provide a baseline for molecular studies on this genus. Integrated Termite Management (7) practices are recommended for the control of this pest species in the orchards.

### 4.1 Data availability and specimen deposition

In response to submitted sequence of *A. dehraensis*, accession number PX423737 was received from the GenBank and specimens are submitted to Insect’s Museum at the Department of Entomology, The University of Agriculture, Peshawar, Pakistan.

## Data Availability

The datasets presented in this study can be found in online repositories. The names of the repository/repositories and accession number(s) can be found in the article/supplementary material.
