# Supplementary material for: Biosystematics of Angulitermes dehraensis in the Northwestern Indomalayan region by integrating morphometrics and distributional data with DNA barcoding
Source: Front Insect Sci. 2025 Oct 24;5:1695789. doi: 10.3389/finsc.2025.1695789 (PMC12593492; doi:10.3389/finsc.2025.1695789)
Supplement: Supplementary file 1 [file DataSheet1.docx]

**Supplementary data**

# DNA extraction and amplification protocol

**Table S1**. DNA extraction and amplification protocol from the Soldier termite of *Angulitermes dehraensis*

| **S. No.** | **Step** |
| --- | --- |
| 1 | Leg tissues was broken with the end of pipette tip in 200.0µl (10%; w/v) of chelex 100 (Walsh et al., 1991) in ddH_2_0 of 0.5ml Eppendorf tube. |
| 2 | Tubes were vortexed and placed at 99.9ºC for 15 mins (using PCR machine) and centrifuged for 2-3 min for induced phase separation. The 2µl upper phase of extracted DNA was used for polymerase chain reaction (PCR). |
| 3 | The PCR was used to amplify ~658 bp fragment of the *COII* gene with the primers A-tLeu CAGATAAGTGCATTGGATTT (forward); B-tLys GTTTAAGAGACCAGTACTTG (reverse) primers (Inward et al. 2007 and Fayle, et al. 2015) by adding 2µl of sample DNA to 23µl master mix in the tubes. |
| 4 | PCR reaction was processed for initial denaturation of five-minute at 94 °C, followed by 35 cycles of 94°C for 10 s, 50°C for 20 s, 72°C for 45 s and 72°C for 07 minutes, and stored at 4°C. |
| 5 | The PCR products were analysed by electrophoresis on 2% agarose gel. For cleaning 2 µl of ExoSAP-IT was added to every 5µl of PCR product (stored in ice) and incubated for 15 min at 37°C and 15 min at 80°C. |

**Table S2.** Morphometrics comparison of *Angulitermes* *dehraensis* with the range in literature for species confirmation

| **S. no.** | **Parameters** | **Range in literature (min)** | **Range in literature (Max)** | **Observed range Mean** | **SD** | **SE** | **CV** |
| --- | --- | --- | --- | --- | --- | --- | --- |
| 1 | Total body length | 3.90 | 4.60 | 4.20 | 0.21 | 0.09 | 5.05 |
| 2 | Head length with mandible | 2.54 | 2.75 | 2.66 | 0.07 | 0.03 | 2.61 |
| 3 | Length of left mandible from the base | 1.34 | 1.45 | 1.43 | 0.04 | 0.02 | 2.86 |
| 4 | Mandible Head Index (Length of mandible/ length of head) | 1.16 | 1.25 | 1.17 | 0.04 | 0.02 | 3.20 |
| 5 | Length of head to side base of mandible | 1.12 | 1.40 | 1.23 | 0.04 | 0.02 | 3.33 |
| 6 | Head width max | 0.89 | 1.06 | 1.04 | 0.18 | 0.08 | 17.23 |
| 7 | Width of pronotum | 0.50 | 0.50 | 0.48 | 0.08 | 0.04 | 17.43 |
| 8 | Postmentom length | 0.43 | 0.45 | 0.44 | 0.02 | 0.01 | 5.28 |
| 9 | Length of labrum | 0.33 | 0.33 | 0.32 | 0.02 | 0.01 | 6.56 |
| 10 | Postmentum max width | 0.30 | 0.30 | 0.30 | 0.01 | 0.01 | 3.85 |
| 11 | Width of labrum | 0.30 | 0.30 | 0.30 | 0.01 | 0.01 | 4.32 |
| 12 | Postmentum median waist | 0.25 | 0.25 | 0.25 | 0.01 | 0.00 | 2.83 |
| 13 | Length of pronotum | 0.23 | 0.23 | 0.23 | 0.01 | 0.00 | 3.82 |
| 14 | Length of frontal projection | 0.10 | 0.13 | 0.11 | 0.01 | 0.00 | 6.43 |
| 15 | Head Index (Width/Length) |  |  | 0.84 | 0.12 | 0.05 | 14.47 |
| 16 | Mandible Head Index (Length of mandible/length of head) |  |  | 1.16 | 0.04 | 0.02 | 3.20 |
| 17 | Pronotum Index (Pronotum Length /Pronotum width) |  |  | 0.50 | 0.09 | 0.04 | 18.76 |
| 18 | Pronotum Head Index (Minimum width of pronotum/Maximum width of head) |  |  | 0.47 | 0.06 | 0.03 | 13.76 |

**Table S3.** Material examined for the morphometric analysis of *Angulitermes dehraensis* from the studied area.

| **S. no** | **District** | **X** | **Y** | **Host food** | **Collector** |
| --- | --- | --- | --- | --- | --- |
| 1 | Haripur | 72.9278 | 33.96878 | Guava | Maid Zaman and Rashid Ali |
| 2 | Haripur | 73.02548 | 33.94733 | Guava | Maid Zaman and Rashid Ali |

**Table S4.** Top 15 accessions from GenBank BLASTn result for Phylogenetic analysis of *Angulitermes dehraensis*.

| **S. no.** | **Accession number** | **Description** | **Max Score** | **Total Score** | **Query Cover** | **E value** | **Per. ident** | **Acc. Len** |
| --- | --- | --- | --- | --- | --- | --- | --- | --- |
| 1 | MZ008538.1 | Amitermes sp. QLD_178 mitochondrion, complete genome | 837 | 837 | 0.99 | 0 | 87.97 | 15922 |
| 2 | MZ008530.1 | Amitermes sp. QLD_088 mitochondrion, complete genome | 832 | 832 | 0.99 | 0 | 87.83 | 15915 |
| 3 | KY224528.1 | Amitermes sp. 5 TB-2017 mitochondrion, complete genome | 837 | 837 | 0.99 | 0 | 87.97 | 14939 |
| 4 | OQ078686.1 | Amitermes unidentatus isolate KE15-134 mitochondrion, partial genome | 859 | 859 | 0.99 | 0 | 88.53 | 15857 |
| 5 | OQ078687.1 | Amitermes unidentatus isolate KE15-141 mitochondrion, partial genome | 854 | 854 | 0.99 | 0 | 88.39 | 16028 |
| 6 | PV057196.1 | Angulitermes sp. isolate KE15-122 mitochondrion, partial genome | 824 | 824 | 0.99 | 0 | 87.75 | 14879 |
| 7 | DQ442073.1 | Angulitermes sp. SA1 cytochrome oxidase subunit II gene, partial cds; mitochondrial | 1280 | 1280 | 1 | 0 | 99.02 | 738 |
| 8 | DQ442165.1 | Microcerotermes dubius cytochrome oxidase subunit II gene, partial cds; mitochondrial | 846 | 846 | 0.99 | 0 | 88.24 | 737 |
| 9 | OQ130293.1 | Microcerotermes subtilis isolate MAD15_148 mitochondrion, partial genome | 833 | 833 | 0.99 | 0 | 87.96 | 15376 |
| 10 | OQ130294.1 | Microcerotermes subtilis isolate MAD15_49 mitochondrion, partial genome | 822 | 822 | 0.99 | 0 | 87.68 | 15377 |
| 11 | DQ442228.1 | Promirotermes cf. redundans cytochrome oxidase subunit II gene, partial cds; mitochondrial | 848 | 848 | 0.99 | 0 | 88.22 | 734 |
| 12 | KP026266.1 | Promirotermes redundans mitochondrion, partial genome | 848 | 848 | 0.99 | 0 | 88.22 | 15215 |
| 13 | OL875039.1 | Promirotermes redundans mitochondrion, partial genome | 848 | 848 | 0.99 | 0 | 88.22 | 15001 |
| 14 | DQ442229.1 | Promirotermes redundans cytochrome oxidase subunit II gene, partial cds; mitochondrial | 832 | 832 | 0.99 | 0 | 87.8 | 738 |
| 15 | KY224554.1 | Promirotermes sp. A TB-2017 mitochondrion, complete genome | 832 | 832 | 0.99 | 0 | 87.8 | 14950 |


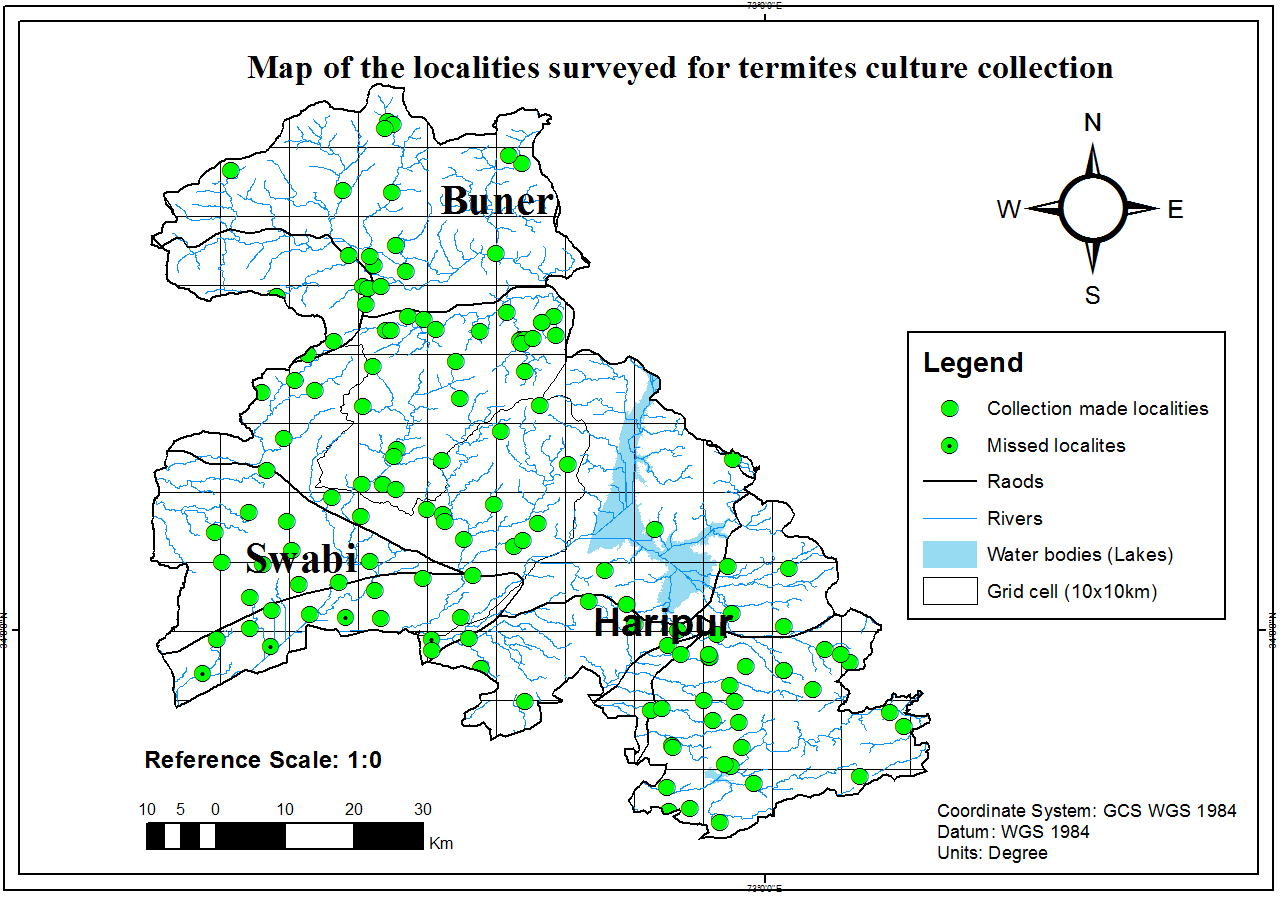


**Figure S1.** Map of the localities surveyed for termite’s culture collection from the selected region of Northwestern Indomalaya region (Pakistan; Khyber Pakhtunkhwa; Swabi, Buneri and Haripur).


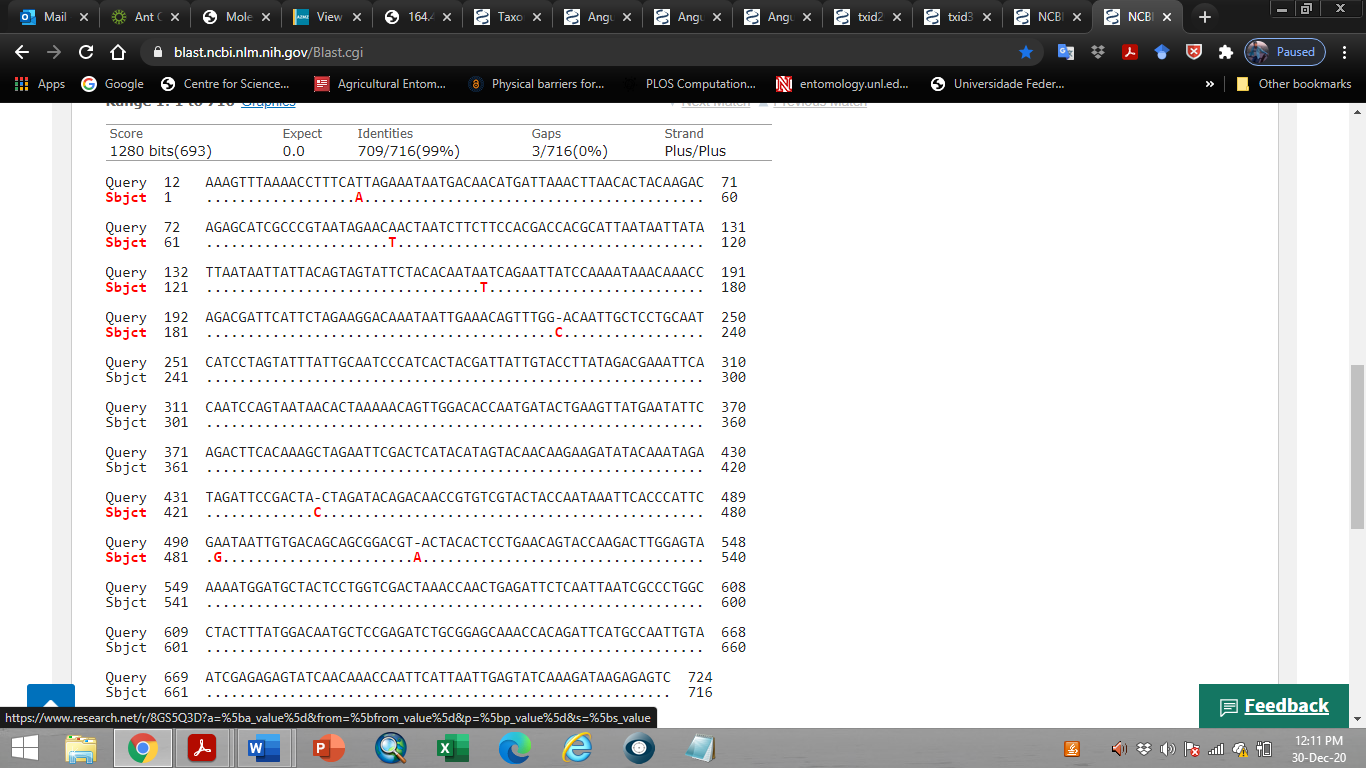


**Figure S2.** Alignment of the *Angulitermes dehriensis* (subject) with the *Angulitermes* sp. (DQ442073.1) (Query) for analogy validation.


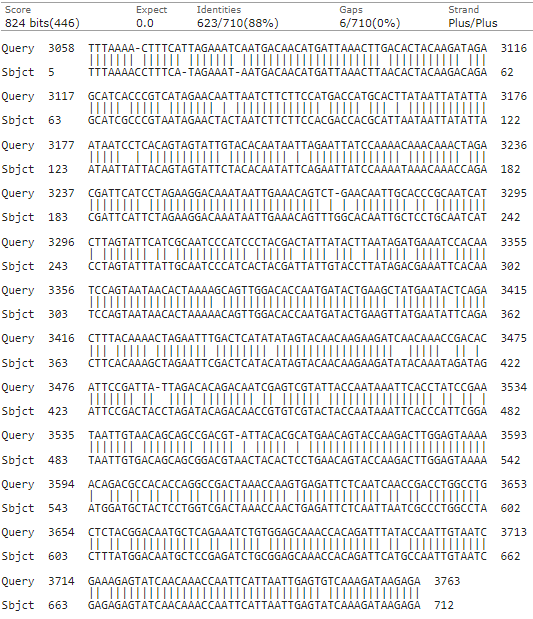


**Figure S3.** Alignment of the *Angulitermes dehriensis* (subject) with the *Angulitermes* sp. (PV057196.1) (Query) for analogy validation.

# Phylogenetic analysis

**N-J tree:**

The evolutionary history was inferred using the Neighbor-Joining method [1]. The optimal tree with the sum of branch length = 0.45288142 is shown. The confidence probability (multiplied by 100) that the interior branch length is greater than 0, as estimated using the bootstrap test (500 replicates is shown next to the branches [2, 3]. The tree is drawn to scale, with branch lengths in the same units as those of the evolutionary distances used to infer the phylogenetic tree. The evolutionary distances were computed using the Kimura 2-parameter method [4] and are in the units of the number of base substitutions per site. The analysis involved 16 nucleotide sequences. All positions containing gaps and missing data were eliminated. There were a total of 700 positions in the final dataset. Evolutionary analyses were conducted in MEGA6 [5].

**M-L tree:**

The evolutionary history was inferred by using the Maximum Likelihood method based on the Tamura-Nei model [6]. The tree with the highest log likelihood (-2573.8283) is shown. The percentage of trees in which the associated taxa clustered together is shown next to the branches. Initial tree(s) for the heuristic search were obtained by applying the Neighbor-Joining method to a matrix of pairwise distances estimated using the Maximum Composite Likelihood (MCL) approach. The tree is drawn to scale, with branch lengths measured in the number of substitutions per site. The analysis involved 16 nucleotide sequences. All positions containing gaps and missing data were eliminated. There were a total of 700 positions in the final dataset. Evolutionary analyses were conducted in MEGA6 [5].

References

1. Saitou N. and Nei M. (1987). The neighbor-joining method: A new method for reconstructing phylogenetic trees. Molecular Biology and Evolution 4:406-425.

2. Dopazo J. (1994). Estimating errors and confidence intervals for branch lengths in phylogenetic trees by a bootstrap approach. Journal of Molecular Evolution 38:300-304.

3. Rzhetsky A. and Nei M. (1992). A simple method for estimating and testing minimum evolution trees. Molecular Biology and Evolution 9:945-967.

4. Kimura M. (1980). A simple method for estimating evolutionary rate of base substitutions through comparative studies of nucleotide sequences. Journal of Molecular Evolution 16:111-120.

5. Tamura K., Stecher G., Peterson D., Filipski A., and Kumar S. (2013). MEGA6: Molecular Evolutionary Genetics Analysis version 6.0. Molecular Biology and Evolution30: 2725-2729.

6. Tamura K. and M., Nei. 1993. Estimation of the number of nucleotide substitutions in the control region of mitochondrial DNA in humans and chimpanzees. Molecular Biology and Evolution 10:512-526.
